# Perinatal urinary benzophenone-3 concentrations and glucose levels among women from a fertility clinic

**DOI:** 10.1186/s12940-020-00598-7

**Published:** 2020-04-28

**Authors:** Zifan Wang, Lidia Mínguez-Alarcón, Paige L. Williams, Andrea Bellavia, Jennifer B. Ford, Myra Keller, John C. Petrozza, Antonia M. Calafat, Russ Hauser, Tamarra James-Todd

**Affiliations:** 1 Department of Epidemiology, Harvard T.H. Chan School of Public Health, 677 Huntington Ave, Boston, MA 02115 USA; 2 Department of Environmental Health, Harvard T.H. Chan School of Public Health, Boston, MA USA; 3 Department of Biostatistics, Harvard T.H. Chan School of Public Health, Boston, MA USA; 4 Vincent Department of Obstetrics and Gynecology, Massachusetts General Hospital, Boston, MA USA; 5 National Center for Environmental Health, Centers for Disease Control and Prevention, Atlanta, GA USA

**Keywords:** Benzophenone-3, Glucose levels, Pregnancy, Endocrine disrupting chemicals, Infertility, Environmental epidemiology

## Abstract

**Background:**

Subfertile women have higher risk of glucose intolerance during pregnancy. Studies suggest associations between several endocrine disrupting chemicals (EDCs) and pregnancy glucose levels. However, the association between benzophenone-3 (BP-3), an EDC widely found in sunscreen, and pregnancy glucose levels remains unclear. We aimed to assess the association between perinatal exposures to BP-3 and pregnancy glucose levels in subfertile women.

**Methods:**

We evaluated 217 women from a prospective cohort based at a fertility clinic who had urinary BP-3 concentrations measured during 3-month preconception, first and/or second trimesters, and blood glucose measured at glucose load tests (GLTs) during late pregnancy. Multivariable linear and logistic regression models were used to assess associations between time-specific BP-3 in quartiles (Q1 – Q4) and mean glucose levels, as well as odds of abnormal GLT (glucose level ≥ 140 mg/dL), adjusting for potential confounders. Effect modification was assessed by age, season, BMI, infertility diagnosis, sex of fetus (es) and physical activity.

**Results:**

Women with higher first trimester BP-3 concentrations had lower mean glucose levels [mean glucose (95% CI) for Q4 vs Q1 = 103.4 (95.0, 112.5) vs. 114.6 (105.8, 124.2) mg/dL]. Women with higher second trimester BP-3 concentrations had lower odds of abnormal GLT [OR (95% CI) for Q3 vs. Q1 = 0.12 (0.01, 0.94)]. The associations between BP-3 and glucose levels were modified by several factors: women with female-factor infertility, urine collected during summer, older age, lower BMI, or carried female fetus (es) had the strongest inverse associations between BP-3 and glucose levels, while no associations were observed in the remaining subgroups.

**Conclusions:**

Time-specific inverse associations between BP-3 and pregnancy glucose levels existed in subfertile women, and especially among certain subgroups of this high-risk-population.

## Background

Gestational diabetes mellitus (GDM) has increased over the past decades, currently affecting 7.6% of pregnant women in the United States [1]. Certain subgroups of the population have even greater risk of GDM, including those who conceived through assisted reproductive techniques (ARTs) compared to women who conceived naturally [2]. Subfertile women have almost double the risk of GDM conditions, such as polycystic ovarian syndrome (PCOS) or metabolic responses to ART (e.g. response to induced ovulation, underlying exacerbated metabolic factors during ART) that may lead to glucose dysregulation in pregnancy [3]. With infertility affecting 10–20% of U.S. couples [4], the increased risk of GDM remains a concern, as it is associated with adverse outcomes including premature delivery, preeclampsia, cesarean delivery, or neonatal hypoglycemia [5]. Interestingly, women with elevated glucose levels, even below the limit of clinical GDM, are at higher risk of these complications [6].

Aside from traditional lifestyle factors [7], there has been growing evidence suggesting that some environmental factors like endocrine disrupting chemicals (EDCs) may also contribute to elevating glucose levels during pregnancy [8]. For example, persistent organic pollutants (POPs) were found to increase GDM risk in average-risk populations [9]. In addition, we previously found associations between pregnancy glucose levels and certain non-persistent chemicals, including bisphenol A [10], certain parabens [11], and certain phthalates [12], in higher-risk (i.e. subfertile) populations from the same study cohort. However, benzophenone-3 (BP-3)—a highly ubiquitous EDC—has not been assessed in relation to glucose levels, particularly in the insulin-resistant period during pregnancy [13].

BP-3 is a derivative of benzophenone-type ultraviolet (UV) blockers. It was detected in over 96% of the U.S. general population between 2003 and 2012 [14] and 100% of a diverse cohort of U.S. pregnant women between 2009 and 2010 [15]. BP-3 is widely used in sunscreen products, as well as other consumer products including shampoos, fragrances, nail polishes, furniture, clothing, carpets, and in plastic products as UV stabilizers [16]. While other EDCs have been found to increase the risk of glucose intolerance through regulating pancreatic β-cell function, inducing insulin resistance, or promoting oxidative stress [17–19], the role of BP-3 in glucose regulation remains understudied. A few epidemiological studies found positive associations between maternal benzophenone biomarkers and regulators for insulin metabolization (e.g. IGF-I and its binding protein IGFBP3) [20] or predictors for diabetes (e.g. markers of systematic inflammation and oxidative stress) [21, 22], yet another case-control study in Saudi Arabia suggested a potential inverse association between BP-3 concentrations and type 2 diabetes [23]. However, the associations between perinatal BP-3 concentrations and pregnancy glucose levels have not been evaluated. It is also questionable how timing of exposure would have any differing effects on glucose levels, as BP-3 is non-persistent [24], and maternal glucose metabolism changes across pregnancy, with increasing insulin resistance during mid- to late-pregnancy [25].

Thus, we aimed to assess the association between time-specific (i.e. preconception, first trimester and second trimester) BP-3 urinary concentrations and blood glucose levels in late pregnancy among subfertile women seeking care at a fertility clinic. We evaluated whether season, maternal age, body mass index (BMI), infertility diagnosis, sex of fetus (es) and physical activity might be potential modifiers of the associations. Understanding the role of this widespread but understudied EDC and its relation to pregnancy glucose levels could provide implications for chemical exposures and GDM risk in a high-risk (i.e. subfertile) subgroup of women.

## Methods

### Study population

This study was conducted among a subset of women participating in the Environment and Reproductive Health (EARTH) Study, an ongoing prospective cohort assessing environmental impact on reproduction based on couples seeking treatment at the Massachusetts General Hospital (MGH) Fertility Center [26]. In this study, we included women who: (1) provided at least one urine sample at preconception (defined as within 3 months prior to conception of the index pregnancy), first trimester (median: 8 weeks gestation), and/or second trimester (median: 22 weeks gestation); (2) had blood glucose levels measured from the 50-g glucose load test (GLT), as a part of the GDM screening test used universally in this cohort; (3) for those who were pregnant more than once during study participation (*n* = 15), only their first pregnancy data was included; (4) had a live birth. Women with a prior history of diabetes at baseline (including medical/self-reported diabetes, or reported to be on diabetes medications upon enrollment) were excluded (*n* = 6). In total, 217 women were included in the study, who provided 833 urine samples (469 samples from 178 women during 3-month preconception, 194 samples from 194 women during the first trimester, and 170 samples from 170 women during the second trimester). The study period ranged from 2009 to 2017. We obtained signed informed consent from all participants. This study was approved by the Partners IRB, Harvard IRB, and the Centers for Disease Control and Prevention (CDC).

### Urine collection and BP-3 quantification

Spot urine samples were collected during preconception, first trimester, and/or second trimester in sterile polypropylene cups. Specific gravity (SG) [27] was measured with a handheld refractometer (National Instrument Company, Inc., Baltimore, MD, USA). Urine samples were divided, frozen (− 20 °C) and stored (− 80 °C) before being transported to the CDC (Atlanta, GA, USA) on dry ice for quantification of BP-3 concentrations. Online solid phase extraction along with high-performance liquid chromatography tandem mass spectrometry was utilized for quantification (detailed description elsewhere [28]). The limit of detection (LOD) was 0.4 ng/mL or 0.2 ng/mL depending on year of analysis.

### Outcome assessment

Blood glucose levels during pregnancy were assessed using a 1-h non-fasting, 50-g glucose load test (GLT) at late-second/early-third trimester (median: 27 weeks gestation) as part of the two-step method with Carpenter-Coustan criteria for GDM screening in this study population. Data were abstracted from medical records. In this study, blood glucose levels were used as: (1) continuous glucose levels from GLT; (2) dichotomized glucose status, where abnormal GLT was defined as glucose level ≥ 140 mg/dL [29], in which women would have been referred for additional GDM screening based on the elevated glucose level.

### Clinical data and covariates assessment

Sociodemographic and lifestyle factors were collected from questionnaires upon enrollment. Weight (kg) and height (m) were measured by trained study staff and body mass index (BMI) was calculated as weight/height^2^. Physician diagnosis of PCOS was abstracted from electronic medical records. Infertility was diagnosed by physicians based on the Society for Assisted Reproductive Technology definitions [i.e. female-factor (including diminished ovarian reserve, endometriosis, uterine, ovulatory, tubal, other), male-factor, or unexplained]. The study included pregnancies conceived by in vitro fertilization (IVF), intrauterine insemination (IUI), or natural conception. Number and sex of fetus (es) per birth were abstracted from maternal delivery records. Dates of urine sample collection and GLT were collected to determine seasonality (winter: Dec-Feb, spring: Mar-May, summer: Jun-Aug, fall: Sep-Nov).

### Statistical analysis

Urinary dilution was accounted for by using SG-adjusted BP-3 concentration (ng/mL) using the formula P_c_ = P [(1.016–1) / (SG – 1)], where P_c_ is the SG-adjusted concentration, P is the measured concentration, and 1.016 is the mean SG concentration for all urine samples in this subset of the study population. Concentrations below the LOD were substituted by a value equal to the LOD divided by square root of 2 before SG-adjustment [30]. During pregnancy, participants provided one urine sample at each trimester. During preconception, 82% (139/170) of the participants had multiple urine samples. Thus, preconception BP-3 concentrations were calculated as the geometric means of SG-adjusted BP-3 concentrations per participant. For each time point, SG-adjusted BP-3 concentrations were categorized into quartiles. The lowest quartile was used as referent category in all analyses. Due to a skewed distribution, blood glucose levels were log-transformed to achieve normality and then back transformed for better interpretability.

Baseline characteristics of the participants in this study were summarized across quartiles of SG-adjusted BP-3 concentrations in the form of mean ± standard deviation (SD) or number (percentages, %), with *p*-values obtained by Kruskal-Wallis test for the continuous variables or Fisher’s exact test for the categorical variables. Time-specific distribution of urinary BP-3 concentrations were summarized by calculating geometric means (95% CIs) and percentiles in the study. As a comparison, we calculated the weighted geometric means (95% CIs) and percentiles for BP-3 concentrations from 2009 to 2016 cycles of the National Health and Nutrition Examination Survey (NHANES) among pregnant women and among women aged 23–47 years (comparable to our study population). To identify potential seasonal patterns of SG-adjusted BP-3 concentrations, geometric means (95% CI) during each season were calculated and compared using linear regression (accounted for repeated measurements).

Multivariable linear regression models were used to identify the association between quartiles of SG-adjusted BP-3 concentrations in separate time periods and log-transformed blood glucose levels, adjusted for potential confounders. Population marginal means of glucose levels were used to reveal the population average glucose levels for each quartile of BP-3 concentrations. The median log SG-adjusted bezophenone-3 concentration in each quartile were applied to the models as a continuous variable to test for linear trend. We also used multivariable logistic regression models to identify the association between time-specific BP-3 concentrations and odds of abnormal GLT, defined as glucose level ≥ 140 mg/dL.

According to a priori knowledge with evidence of being risk factors for GDM [31, 32] and potentially associated with BP-3, we posited that maternal age, race/ethnicity, education level, physical activity, smoking status, family history of diabetes, seasonality, maternal BMI, PCOS status, number of fetus, sex of fetus (es) and infertility diagnosis and treatment were potential confounders. Models included the following covariates: maternal age (years), pre-pregnancy BMI (< 25 kg/m^2^, ≥25 kg/m^2^), family history of diabetes (yes, no), baseline smoking status (never, ever), total physical activity (hours/week), race (white, non-white), education level (college graduate or higher, other), infertility diagnosis (male-factor, female-factor, unexplained), physician-diagnosed PCOS (yes, no), infertility treatment (IVF, IUI, natural), fetus number (1, ≥2), sex of fetus (es) (male, female or unknown) and season of urine collection (spring, summer, fall, winter). We also evaluated potential effect modification by season (summer versus other), maternal age (< 37 versus ≥37 years), maternal BMI (< 25 versus ≥25 kg/m^2^), infertility diagnosis (male-factor, female-factor, versus unexplained), sex of fetus (es) (male versus female), and physical activity (< 5.5 versus ≥5.5 h/week) by adding interaction terms with BP-3 in multivariable models, as well as conducting stratified analyses.

To determine the robustness of results, we performed several sensitivity analyses. First, given the seasonal pattern of BP-3 concentrations in our study, we evaluated associations with and without seasonality in the model. Since outdoor activity may be associated with sunscreen use and glucose levels, we also further adjusted for moderate/heavy outdoor work. We then included time difference between the date of urine collection and GLT in the models. Since the collection of preconception urine samples preceded determination of the sex of fetus (es), to assess any potential mediation by sex, we also evaluated associations between preconception BP-3 and glucose without adjusting for sex of fetus (es). Moreover, we excluded urine samples collected on the same day of GLT (*n* = 10) and repeated the analyses to address potential issues of temporality. Lastly, we averaged BP-3 concentrations from the first and second trimesters and repeated the analyses. All statistical analyses were performed using SAS version 9.4 (SAS Institute Inc., Cary, NC). Two-sided *p*-values < 0.05 were considered statistically significant unless otherwise specified.

## Results

Baseline characteristics of the study population were summarized in Table 1. Participants had an average age of 34.8 years. Most were non-Hispanic white (82%), never-smokers (76%), and college graduates or higher (86%). 66% had BMI less than 25 kg/m^2^, 44% had unexplained infertility at baseline, and 60% underwent IVF treatment. The majority delivered a singleton (80%), and 52% delivered female fetus (es). The mean blood glucose level from the GLT was 116.3 mg/dL (SD: 28.3). In general, baseline characteristics were not significantly different comparing the highest quartile of BP-3 to the lowest quartile, except for infertility diagnosis and time difference between urine collection and GLT, where women in the highest quartile of first trimester BP-3 were less likely to be diagnosed with male-factor infertility, and those in the highest quartile of first/second trimester BP-3 tended to have longer time differences between urine collection and GLT.

**Table 1:** Baseline characteristics among 217 women in the EARTH Study by BP-3 quartiles (Q4 versus Q1)

Characteristic	Total *n* = 217	Preconception *n* = 178^a^			First Trimester *n* = 194^b^			Second Trimester *n* = 170^c^		
		BP-3 Q1	BP-3 Q4	*p*-value^d^	BP-3 Q1	BP-3 Q4	*p*-value^d^	BP-3 Q1	BP-3 Q4	*p*-value^d^
N of participants	217	44	44		48	48		42	42	
Age at pregnancy (years)				0.38			0.29			0.14
Mean ± SD	34.8 ± 3.8	34.9 ± 4.1	34.2 ± 3.5		35.1 ± 4.2	34.2 ± 3.7		35.1 ± 4.2	33.8 ± 3.3	
Range	23–47	25–42	27–41		23–47	27–43		23–47	27–41	
Pre-pregnancy BMI (kg/m^2^)				0.50			1.00			0.66
BMI < 25	144 (66.4)	27 (61.4)	31 (70.5)		31 (64.6)	32 (66.7)		26 (61.9)	23 (54.8)	
BMI ≥ 25	73 (33.6)	17 (38.6)	13 (29.6)		17 (35.4)	16 (33.3)		16 (38.1)	19 (45.2)	
Family history of DM	28 (12.9)	6 (13.6)	5 (11.4)	1.00	6 (12.5)	4 (8.3)	0.74	4 (9.5)	6 (14.3)	0.74
Smoking status				0.57			0.84			0.09
Never smoked	165 (76.0)	38 (86.4)	35 (79.6)		38 (79.2)	39 (81.3)		32 (76.2)	32 (76.2)	
Former smoker	45 (20.7)	6 (13.6)	9 (20.5)		9 (18.8)	7 (14.6)		10 (23.8)	6 (14.3)	
Current smoker	7 (3.2)	0 (0.0)	0 (0.0)		1 (2.1)	2 (4.2)		0 (0.0)	4 (9.5)	
Total physical activity (hours/week)	7.9 ± 10.0	6.4 ± 7.9	7.5 ± 5.8	0.11	7.7 ± 7.9	6.5 ± 5.2	0.74	9.0 ± 12.6	7.9 ± 6.9	0.45
Race				0.72			0.47			1.00
Non-Hispanic White	178 (82.0)	34 (77.3)	38 (86.4)		39 (81.3)	37 (77.1)		34 (81.0)	33 (78.6)	
Black/African American	6 (2.8)	3 (6.8)	1 (2.3)		0 (0.0)	3 (6.3)		2 (4.8)	1 (2.4)	
Asian	20 (9.2)	4 (9.1)	3 (6.8)		5 (10.4)	4 (8.3)		3 (7.1)	4 (9.5)	
Other	13 (6.0)	3 (6.8)	2 (4.6)		4 (8.3)	4 (8.3)		3 (7.1)	4 (9.5)	
Education level				0.12			0.91			1.00
High school graduate or less	2 (0.9)	0 (0.0)	0 (0.0)		1 (2.1)	1 (2.1)		0 (0.0)	1 (2.4)	
Some college	9 (4.2)	3 (6.8)	0 (0.0)		2 (4.2)	1 (2.1)		2 (4.8)	1 (2.4)	
College graduate or higher	186 (85.7)	34 (77.3)	40 (90.9)		40 (83.3)	39 (81.3)		35 (83.3)	35 (83.3)	
Missing	20 (9.2)	7 (15.9)	4 (9.1)		5 (10.4)	7 (14.6)		5 (11.9)	5 (11.9)	
Infertility diagnosis				0.73			0.04			0.24
Male-factor	56 (25.8)	14 (31.8)	11 (25.0)		16 (33.3)	6 (12.5)		17 (63.0)	10 (37.0)	
Female-factor	66 (30.4)	11 (25.0)	11 (25.0)		12 (25.0)	20 (41.7)		9 (21.4)	13 (31.0)	
Unexplained	95 (43.8)	19 (43.2)	22 (50.0)		20 (41.7)	22 (45.8)		16 (38.1)	19 (45.2)	
Fetus number of the pregnancy				0.78			0.77			0.80
1	173 (79.7)	35 (79.6)	36 (81.8)		40 (83.3)	42 (87.5)		31 (73.8)	33 (78.6)	
2	42 (19.4)	9 (20.5)	7 (15.9)		8 (16.7)	6 (12.5)		11 (26.2)	9 (21.4)	
≥ 3	2 (0.9)	0 (0.0)	1 (2.3)		0 (0.0)	0 (0.0)		0 (0.0)	0 (0.0)	
Infertility treatment				0.58			0.58			0.64
IVF	130 (59.9)	22 (50.0)	26 (59.1)		27 (56.3)	26 (54.2)		25 (53.2)	22 (46.8)	
IUI	41 (18.9)	12 (27.3)	8 (18.2)		11 (22.9)	8 (16.7)		8 (19.1)	12 (28.6)	
Natural conception	46 (21.2)	10 (22.7)	10 (22.7)		10 (20.8)	14 (29.2)		9 (21.4)	8 (19.1)	
Physician-diagnosed PCOS	13 (6.0)	5 (11.4)	2 (4.6)	0.43	2 (4.2)	6 (12.5)	0.27	1 (2.4)	4 (9.5)	0.36
Sex of fetus (es)				0.39			0.31			1.00
Male	102 (47.0)	21 (47.7)	16 (36.4)		21 (43.8)	26 (54.2)		20 (47.6)	20 (47,6)	
Female	112 (51.6)	23 (52.3)	27 (61.4)		27 (56.3)	21 (43.8)		22 (52.4)	21 (50.0)	
Missing	3 (1.4)	0 (0.0)	1 (2.3)		0 (0.0)	1 (2.1)		0 (0.0)	1 (2.4)	
Season of urine collection	/			0.82			0.30			0.07
Winter		7 (15.9)	5 (11.4)		12 (25.0)	8 (16.7)		16 (38.1)	6 (14.3)	
Spring		13 (29.6)	11 (25.0)		17 (35.4)	13 (27.1)		8 (19.1)	10 (23.8)	
Summer		9 (20.5)	12 (27.3)		7 (14.6)	14 (29.2)		10 (23.8)	18 (42.9)	
Fall		15 (34.1)	16 (36.4)		12 (25.0)	13 (27.1)		8 (19.1)	8 (19.1)	
Time differences between BP-3 and glucose measurements (days)	/	200.0 ± 29.5	204.5 ± 35.0	0.63	128.9 ± 14.7	138.0 ± 16.2	0.003	39.2 ± 19.8	47.6 ± 19.2	0.04

Data presented as mean ± SD for continuous variables, or n (%) for categorical variables

Abbreviations: *BP-3* benzophenone-3, *DM* diabetes mellitus, *EARTH*, Environmental and Reproductive Health, *PCOS* polycystic ovarian syndrome, *Q1* quartile 1, *Q4* quartile 4, *IVF* in vitro fertilization, *IUI* intra uterine insemination

^a^ 178 women provided urine samples collected during preconception (defined as within 3 months prior to conception)

^b^ 194 women provided urine samples collected during first trimester

^c^ 170 women provided urine samples collected during second trimester

^d^ P-values obtained by using Kruskal–Wallis test or Fisher’s exact test, for continuous or categorical variables respectively

The distribution of time-specific BP-3 concentrations was shown in Table 2. Detection frequencies were 99.6%, 99.5% and 98.8% for preconception, first trimester, and second trimester, respectively. Overall, the geometric means of SG-adjusted BP-3 concentrations were similar across time windows [geometric mean (95% CI) in ng/mL = 166.6 (130.1, 213.4), 142.4 (109.5, 185.2), 189.1 (139.4, 256.5), respectively]. Compared to the general U.S. population from the 2009–2016 NHANES database which included only pregnant women (BP-3 geometric mean = 42.9 ng/mL) and women with similar age to our study regardless of pregnancy status (BP-3 geometric mean = 39.4 ng/mL), the concentrations in our study population were much higher (See additional file: Table S1). Because sunscreen is the most common source of BP-3, whose use may be greatly dependent on season, the distributions of BP-3 concentrations across seasons were calculated (see Additional file 1: Figure S1). The geometric means (95% CI) in ng/mL of SG-adjusted BP-3 concentrations during summer [286.7 (203.2, 404.6)] were significantly higher than winter [120.3 (92.3, 156.8)] in all urine samples in this study population (*p*-value < 0.0001).

**Table 2:** Time-specific distribution of urinary BP-3 concentrations (ng/mL) during the perinatal period for 217 women in the EARTH Study

	N^a^	Detection %	Geometric mean (95% CI)	Percentile						
				Min	25th	50th	75th	90th	95th	Max
Urine samples collected during preconception (mean preconception BP-3 exposure for each participant)										
BP-3	178	99.6%	131.6 (102.5, 168.9)	<LOD	50.4	118.3	431.3	1318.3	2469.3	9000.0
SG-adjusted BP-3			166.6 (130.1, 213.4)	<LOD	58.7	153.6	494.5	1710.3	2370.2	8313.8
Urine samples collected during the first trimester (one sample per participant)										
BP-3	194	99.5%	106.4 (81.7, 138.6)	<LOD	29.8	78.5	364.7	1304.2	3397.7	9115.3
SG-adjusted BP-3			142.4 (109.5, 185.2)	<LOD	39.0	124.6	500.3	1710.2	3818.3	7888.0
Urine samples collected during the second trimester (one sample per participant)										
BP-3	170	98.8%	132.4 (95.8, 182.9)	<LOD	33.0	130.9	667.0	1990.0	4577.2	21,728.9
SG-adjusted BP-3			189.1 (139.4, 256.5)	<LOD	40.1	169.1	811.8	2960.5	5358.2	17,383.1

Abbreviations: *BP-3* benzophenone-3, *LOD* limit of detection (0.4 ng/ml for some years and 0.2 ng/ml for other years)

For concentrations below the LOD: concentrations were assigned a value equal to the LOD divided by square root of 2 for the calculation of geometric means

^a^ Number of participants

In Table 3, we present the association between time-specific BP-3 concentrations and pregnancy glucose levels. We did not observe any significant association between preconception or second trimester BP-3 concentrations and glucose levels (preconception: p-trend = 0.29; second trimester: p-trend = 0.28). However, we found that when assessing BP-3 concentrations during the first trimester and glucose levels, the adjusted average glucose levels among women in the highest BP-3 quartile were significantly lower than that among the lowest BP-3 quartile (103.4 versus 114.6 mg/dL, *p*-value = 0.02, p-trend = 0.07).

**Table 3:** Quartiles of time-specific BP-3 urinary concentrations and blood glucose levels during pregnancy among 217 women in the EARTH Study

Quartile (range) of SG-adjusted BP-3 concentration in ng/mL	Population means of blood glucose level in mg/dL (95% CI) across quartiles of SG-adjusted BP-3 concentrations	
	Unadjusted	Adjusted^a^
Urine samples collected during preconception (mean preconception BP-3 exposure for each participant) (*n* = 178)		
Q1 (<LOD to 58.7)	114.0 (106.4, 122.1)	111.9 (103.0, 121.6)
Q2 (58.7 to 153.3)	113.9 (106.4, 121.9)	113.7 (103.5, 124.9)
Q3 (153.8 to 494.5)	112.5 (105.1, 120.4)	107.1 (97.2, 118.0)
Q4 (498.9 to 8313.8)	109.5 (102.2, 117.3)	108.0 (98.0, 118.9)
*P*-trend^b^	0.38	0.29
Urine samples collected during first trimester (one sample per participant) (*n* = 194)		
Q1 (<LOD to 35.2)	116.4 (108.9, 124.3)	114.6 (105.8, 124.2)
Q2 (39.0 to 124.0)	112.9 (105.8, 120.5)	111.9 (102.6, 121.9)
Q3 (125.3 to 500.3)	121.3 (113.7, 129.5)	118.9 (109.1, 129.4)
Q4 (502.6 to 7888.0)	104.0 (97.4, 111.1)^c^	103.4 (95.0, 112.5)^c^
*P*-trend^b^	0.06	0.07
Urine samples collected during second trimester (one sample per participant) (*n* = 170)		
Q1 (<LOD to 36.3)	115.8 (108.2, 123.9)	110.0 (100.9, 119.9)
Q2 (40.1 to 167.5)	107.2 (100.2, 114.7)	105.3 (96.2, 115.2)
Q3 (170.7 to 811.8)	107.7 (100.7, 115.2)	103.2 (94.4, 112.8)
Q4 (855.2 to 17,383.1)	113.0 (105.5, 120.9)	104.9 (95.7, 114.9)
*P*-trend^b^	0.70	0.28

Abbreviations: *BP-3* benzophenone-3, *LOD* limit of detection, *Q* quartile

^a^ Adjusted for maternal age at pregnancy (years), pre-pregnancy BMI (< 25 kg/m^2^, ≥25 kg/m^2^), family history of diabetes (yes, no), baseline smoking status (never, ever), total physical activity (hours/week), race (white, non-white), education level (college graduate or higher, other), infertility diagnosis (male-factor, female-factor, unexplained), physician-diagnosed PCOS (yes, no), type of infertility treatment (IVF, IUI, natural), fetus number in a pregnancy (1, ≥2), sex of fetus (es) (male, female or missing) and season (spring, summer, fall, winter)

^b^ Test for linear trend were performed using the median SG-adjusted urinary BP-3 concentration in each quartile as a continuous variable in the model

^c^ p-value for comparison against Q1 is < 0.05 (p-value = 0.02 in both unadjusted and adjusted models)

When assessing the association between quartiles of BP-3 and odds of abnormal GLT, there were no significant associations between preconception or first trimester BP-3 concentrations (p-trend = 0.49 and 0.28 respectively) and odds of abnormal GLT. When comparing higher quartile of second trimester BP-3 to the lowest quartile, Q3 vs. Q1 during second trimester was associated with lower odds of abnormal GLT [odds ratio (95% CI) = 0.12 (0.01, 0.94), *p*-value = 0.04), despite the overall non-significant trend (p-trend = 0.90) and no significant differences between Q4 vs. Q1 [odds ratio (95% CI) = 1.04 (0.25, 4.42)].

**Fig. 1:**
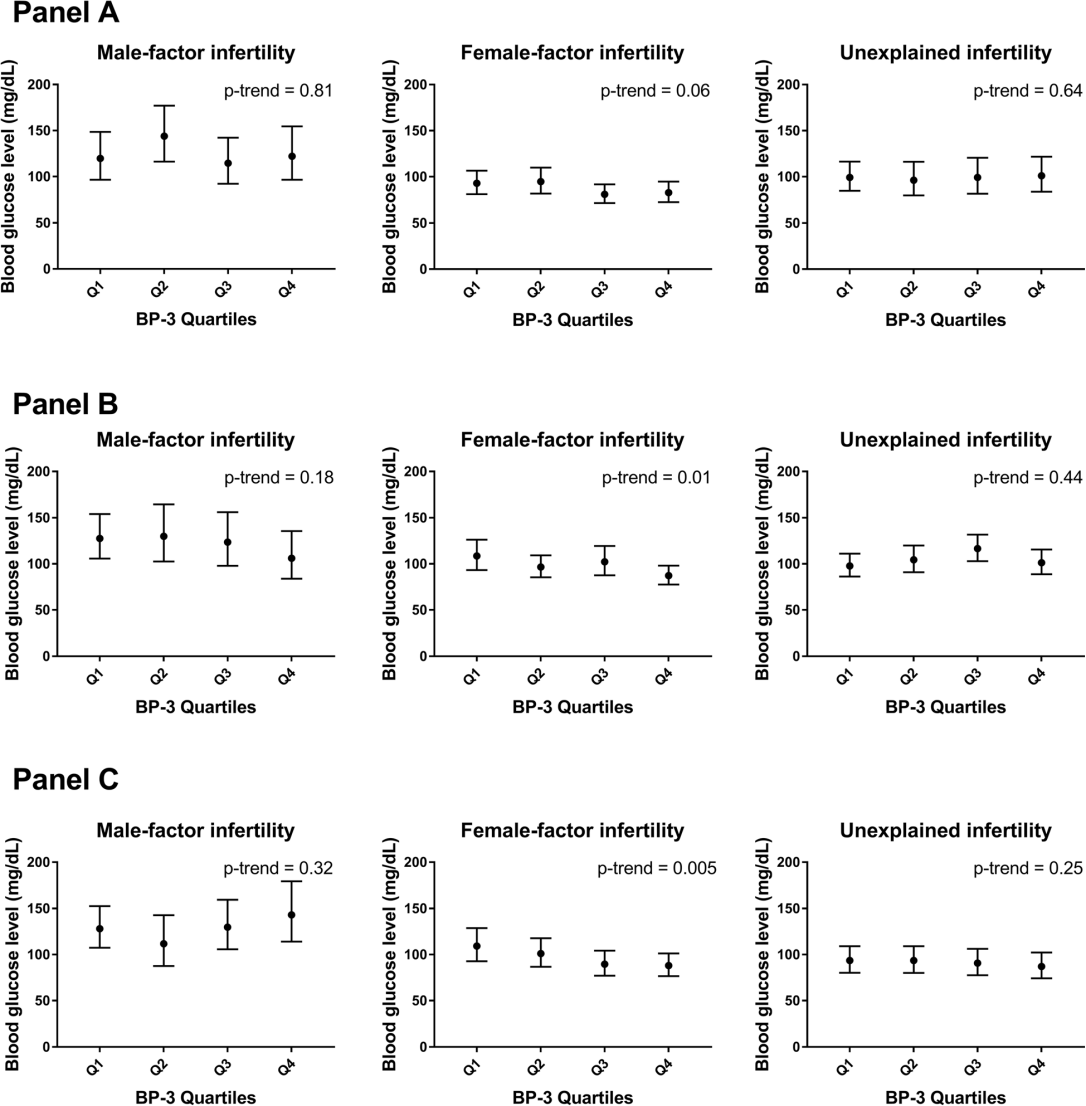
Time-specific SG-adjusted urinary BP-3 concentrations and blood glucose levels, stratified by infertility diagnosis. Panel A: preconception SG-adjusted BP-3 concentrations and blood glucose levels, stratified by infertility diagnosis. Panel B: first trimester SG-adjusted BP-3 concentrations and blood glucose levels, stratified by infertility diagnosis. Panel C: second trimester SG-adjusted BP-3 concentrations and blood glucose levels, stratified by infertility diagnosis. Abbreviations: BP-3: benzophenone-3. Q1-Q4: quartiles of SG-adjusted BP-3 concentrations. All analyses adjusted for maternal age at pregnancy (years), pre-pregnancy BMI (< 25 kg/m^2^, ≥25 kg/m^2^), family history of diabetes (yes, no), baseline smoking status (never, ever), total physical activity (hours/week), race (white, non-white), education level (college graduate or higher, other), physician-diagnosed PCOS (yes, no), type of infertility treatment (IVF, IUI, natural), fetus number in a pregnancy (1, ≥2), sex of fetus (es) (male, female or missing), and season (spring, summer, fall, winter). Test for linear trend (p-trend) were performed using the median SG-adjusted urinary BP-3 concentration in each quartile as a continuous variable in the model

**Fig. 2:**
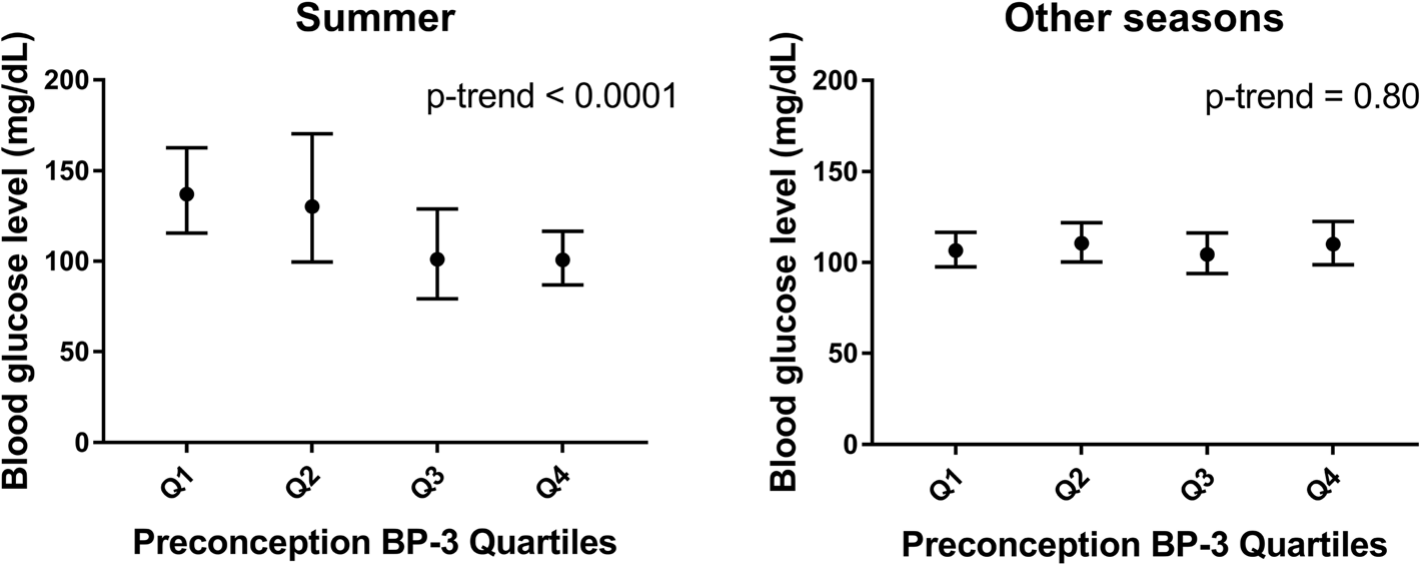
Preconception SG-adjusted urinary BP-3 concentrations and blood glucose levels, stratified by season of urine collection. Abbreviations: BP-3: benzophenone-3. Q1-Q4: quartiles of SG-adjusted BP-3 concentrations. Season of urine collection: summer vs. other seasons. Adjusted for maternal age at pregnancy (years), pre-pregnancy BMI (< 25 kg/m^2^, ≥25 kg/m^2^), family history of diabetes (yes, no), baseline smoking status (never, ever), total physical activity (hours/week), race (white, non-white), education level (college graduate or higher, other), infertility diagnosis (male-factor, female-factor, unexplained), physician-diagnosed PCOS (yes, no), type of infertility treatment (IVF, IUI, natural), fetus number in a pregnancy (1, ≥2), and sex of fetus (es) (male, female or missing). Test for linear trend were performed using the median SG-adjusted urinary BP-3 concentration in each quartile as a continuous variable in the model

For potential heterogeneity of these associations, we observed effect modification by infertility diagnosis for first and second trimester BP-3 concentrations and glucose levels (p for interaction = 0.03 and 0.08, respectively). Specifically, we observed inverse associations between BP-3 concentrations in all time windows and glucose levels only among those with female-factor infertility (p-trend = 0.06, 0.01, and 0.005 during preconception, first and second trimester respectively, Fig. 1, see Additional file 1: Table S2). We also found effect modification by season of urine collection for the association between preconception BP-3 and blood glucose level (p for interaction = 0.005, Fig. 2, see Additional file 1: Table S3), where preconception BP-3 concentrations in urine samples collected during summer were inversely associated with blood glucose levels (p-trend < 0.0001) but not for other seasons. Associations were not significantly modified (p for interaction > 0.10) by age, BMI, sex of fetus (es) (see Additional file 1: Table S4 – S6), or physical activity (data not shown), but in stratified analyses there were inverse associations between glucose levels and: (1) preconception BP-3 among women aged ≥37 (p-trend = 0.04); (2) first trimester BP-3 among women with BMI < 25 kg/m^2^ (p-trend = 0.03); and (3) first trimester BP-3 among women with female fetus (es) (p-trend = 0.009).

The robustness of results was evaluated through sensitivity analyses (data not shown). First, excluding seasonality or sex of fetus (es), or including outdoor work, year of urine collection, and time difference from urine collection to GLT in the multivariable models had very little impact on all results. Secondly, when excluding urine samples collected on the same day of GLT to address for temporality, similar patterns of glucose levels across BP-3 quartiles remained. Lastly, when assessing average BP-3 concentrations from women with both first and second trimesters samples (*n* = 153), the adjusted average glucose levels among those in the highest BP-3 quartile (99.2 mg/dL) were still significantly lower than in the lowest quartile (110.1 mg/dL, *p*-value = 0.04), with a significant linear trend across quartiles (p-trend = 0.04).

## Discussion

In this study population of subfertile women, we found time-specific inverse associations between BP-3 urinary concentrations and blood glucose levels measured at late pregnancy. While higher first trimester BP-3 concentrations were associated with significantly lower mean glucose levels, preconception and second trimester BP-3 concentrations did not show this association with glucose levels. Interestingly, some negative associations were stronger among women with female-factor infertility, urine samples collected during summer, older age, lower BMI, or carried female fetus (es). Our results suggest that exposure to BP-3 might potentially decrease glucose levels during pregnancy among women seeking infertility treatment.

There have been few epidemiological studies evaluating exposures to BP-3 and diabetes-related outcomes. A case-control study in Saudi Arabia found that the urinary geometric mean concentrations of BP-3 were 4.2 times greater in the non-diabetic control group compared to the type 2 diabetes group [23], which is consistent with our findings of inverse associations between BP-3 and glucose levels. However, this study focused on type 2 diabetes in the general population instead of GDM or pregnancy glucose levels, and their sample size was relatively small (54 cases and 47 controls). One study from Denmark found positive associations between a benzophenone derivative and IGF-I and binding protein IGFBP3 among women pregnant with male fetuses [20], suggesting the potential for benzophenone to regulate insulin metabolism. However, they did not directly measure GDM or glucose levels as outcomes, and BP-3 was measured in serum. Another study from Puerto Rico showed an inverse association between BP-3 and c-reactive protein [21], a marker for systematic inflammation commonly perceived as a predictor for diabetes [33]; but again, their outcome was not glucose levels or diabetes, and the study was cross-sectional.

Several epidemiological studies examined the association between other EDCs and GDM or pregnancy glucose levels. Serum POPs concentrations were found to be positively associated with GDM risk [9]. In addition, previous studies conducted among pregnant women from the EARTH Study showed that urinary bisphenol A was positively associated with glucose levels [10], urinary butylparaben was positively while propylparaben inversely associated with glucose levels [11], and urinary phthalates also showed associations with glucose levels in mixed directions where a positive association was seen between monoethyl phthalate and glucose but an inverse association was seen between mono-isobutyl phthalate and glucose [12]. The association between BP-3 concentrations and pregnancy glucose levels, however, remains unclear. As such, additional work on this aspect is needed to increase the understanding of potential impact of another highly ubiquitous EDC on GDM risk factors.

Our results showed inverse associations between time-specific BP-3 and pregnancy glucose levels. Potential biological mechanisms for the effect of BP-3 on glucose remain unclear and inconsistent. Conflicting animal studies demonstrated that administration of benzophenone led to either decreased or increased glucose levels among rats [34, 35]. One possible mechanism may involve the reproductive-hormone-like potency of BP-3. BP-3 has been reported to possess estrogenic and anti-androgenic effects, but anti-estrogenic activities were also reported with inverse associations between mixtures of BP-3 and benzophenone-1 and estradiol levels among healthy premenopausal women [36, 37]. Moderate estrogens are known to increase insulin sensitivity, but estrogens at supraphysiological levels may induce insulin resistance [38]. As such, it is still unclear in which direction BP-3 would act on the estrogen levels among pregnant women in late pregnancy. Future studies are needed to help identify the potential effect of BP-3 on estrogen and glucose levels among pregnant women. Another possible mechanism may involve thyroid hormone regulations. Triiodothyronine (T3) was found to be positively associated with risk factors for GDM [39]. An animal study indicated that BP-3 could down-regulate genes related to thyroid stimulating hormones with significant decreases in T3 levels [40], and similar associations were found among pregnant women [41]. Therefore, it is possible that exposure to BP-3 could lead to decreased level of T3 and decreased GDM risk, potentially via decreasing endogenous glucose production and plasma glucose [42]. Again, future studies are needed to explore these proposed mechanisms. Studies have also shown borderline associations between BP-3 and enhanced glutathione peroxidase activity [19], which might lead to enhanced anti-inflammatory response and prevention of β-cell dysfunction [43, 44].

We found significant effect modification by infertility diagnosis, where BP-3 concentrations were inversely associated with blood glucose levels specifically among those diagnosed with female-factor infertility. While this finding could be due to chance given the multiple comparisons, it is also possible that some intrinsic mechanisms of BP-3 interacting with infertility might lead to reduced risk of GDM, including potential reductions in the levels of inflammatory markers (e.g. CRP or reactive oxygen species [45]) for pre-existing inflammation related to infertility. More research is needed to understand how BP-3 may act differently on glucose levels according to fertility conditions.

Although we found year-round high urinary concentrations of BP-3, they were especially high during summer, and interestingly the association between BP-3 concentrations and glucose levels were stronger during summer. In two other studies including women from the same study cohort, urinary BP-3 concentrations were positively associated with sunscreen use during the past 24 h [46], and sunscreen use was found to be inversely associated with glucose levels during pregnancy [47], which further strengthened our results as sunscreen is a major source of BP-3. The effect modification by season might suggest unmeasured confounding of sun exposure and vitamin D levels. It is possible that women with higher BP-3 concentrations tend to apply more sunscreens because of increased outdoor activity, therefore gaining more sun exposure that could impact levels of vitamin D in the body, while higher early pregnancy vitamin D levels were shown to be associated with decreased GDM risk [48]. This may also be the reason why we saw inverse associations in women with lower BMI, as this subgroup could spend more time outdoors and have a healthier lifestyle, but we are uncertain why the inverse associations were stronger in women with older age. Due to limitations of our study, we were unable to evaluate this in greater detail and more studies are needed to understand the impact of time spent outdoors, vitamin D levels, and vitamin supplements on associations between BP-3 and glucose levels. In sex-stratified analyses, an inverse association between first trimester BP-3 concentrations and glucose levels was found specifically among women who carried female fetus (es). Although still subject to sample size limitation, this may still point to potential sex-specific effects of BP-3 during pregnancy, possibly through a joint effect of BP-3 and fetus sex on glucose regulation [49, 50]. Further research is needed to identify possible drivers of sex-specific effects of BP-3 during pregnancy.

Our study has several strengths. First, our study is the first to report the associations between BP-3 and glucose levels during pregnancy in a modest-sized subfertile cohort, with measures of BP-3 in multiple time periods before and during pregnancy to evaluate potential sensitive time windows of exposure. Secondly, for preconception, we averaged multiple urine samples for women who had more than one sample, which accounted for possible variability in the exposure measurement. Thirdly, we accounted for a diversity of potential confounders, including lifestyle factors, seasonality, and reproductive conditions, as well as evaluated possible effect modifiers. Fourthly, we conducted sensitivity analysis removing urine samples collected on the same day of GLT, to limit the possibility of reverse causation through a prospective design. Finally, we evaluated this association in a subfertile population at high risk of developing GDM.

Despite these strengths several limitations exist. First, urine samples were convenience samples during clinical visits. Therefore, we cannot rule out the possibility of non-differential exposure misclassification that may bias results toward the null. However, we found in sensitivity analysis that average pregnancy BP-3 concentrations (i.e. geometric mean from first and second trimesters) had similar inverse associations with glucose levels as seen at each individual time period. Second, we were unable to evaluate clinical diagnosis of GDM as an outcome since only 6 women had this condition. Third, due to limited sample size, the results in stratified analyses may be due to chance and should be interpreted with caution. Fourth, our study was based on a high-risk population of women seeking fertility treatment; therefore, our results may not be generalizable to healthier women without fertility problems. Also, BP-3 concentrations in our study population were much higher than pregnant women in a representative subset of the U.S. population during comparable time periods. Even the lowest quartile in our study would likely represent relatively higher concentrations in the general U.S. population. Therefore, we may not be able to capture the full dose-response relationship, specifically between very low BP-3 concentrations and glucose levels. One possible explanation for such big differences in urinary BP-3 concentrations may be differences in the proportion of non-Hispanic white women between both study populations (our study ~ 80% vs. NHANES ~ 40%) [51]. However, demographic characteristics in our study are comparable to nationwide fertility clinics [10], providing insights to women with a higher baseline risk of GDM due to fertility issues. Lastly, residual (e.g. misclassification of infertility diagnosis) or unmeasured confounding (e.g. by dietary factors, outdoor activities, or vitamin D levels) is still possible. Further studies are needed to replicate, as well as account for more detailed nutritional/behavioral factors.

## Conclusion

In conclusion, time-specific associations between higher urinary BP-3 concentrations and lower glucose levels were found among subfertile women. Associations were stronger among women with female-factor infertility, urine samples collected during summer, older age, lower BMI, or carried female fetus (es). While future studies are needed for further understanding of these associations, our results suggest that BP-3, an EDC with ubiquitous existence in consumer products, might affect glucose levels during pregnancy among a group of women at higher risk of GDM.

## Supplementary information

**Additional file 1: Table S1**. Distribution of urinary BP-3 concentrations (ng/mL) among NHANES women during 2009–2016. Legend: Abbreviations: BP-3, benzophenone-3; LOD, limit of detection (0.4 ng/ml in NHANES 2009–2016). All estimates incorporated NHANES multistage sub-sample weights for combined survey cycles. BP-3 concentrations below LOD were assigned a value equal to the LOD divided by square root of 2 in NHANES.^a^ Women in NHANES 2009–2016 who had positive lab pregnancy test results or self-reported pregnant at the time of urine sample collection, with available BP-3 concentrations. Due to disclosure risks, pregnancy status was only released for women 20–44 years of age since 2007.^b^ Women in NHANES 2009–2016 who were 23–47 years old, regardless of pregnancy status. **Table S2.** Quartiles of time-specific BP-3 urinary concentrations and blood glucose levels among pregnant women in the EARTH Study: stratified by infertility diagnosis. Legend: Abbreviations: LOD, limit of detection (0.4 ng/ml for some years and 0.2 ng/ml for other years). *Adjusted for maternal age at pregnancy (years), pre-pregnancy BMI (< 25 kg/m2, ≥25 kg/m2), family history of diabetes (yes, no), baseline smoking status (never, ever), total physical activity (hours/week), race (white, non-white), education level (college graduate or higher, other), physician-diagnosed PCOS (yes, no), fetus number in a pregnancy (1, ≥2), sex of fetus (es) (male, female or missing) and season (spring, summer, fall, winter). † Test for linear trend were performed using the median SG-adjusted urinary BP-3 concentration in each quartile as a continuous variable in the model, adjusted for the above covariates. ‡ Adjusted p for interaction was obtained by adding interaction terms of infertility diagnosis*the median SG-adjusted urinary BP-3 concentration in each quartile to the models described in †. § *p*-value for comparison against Q1 is < 0.05. **Table S3.** Quartiles of time-specific BP-3 urinary concentrations and blood glucose levels among pregnant women in the EARTH Study: stratified by season of urine collection. Legend: Abbreviations: LOD, limit of detection (0.4 ng/ml for some years and 0.2 ng/ml for other years). *Adjusted for maternal age at pregnancy (years), pre-pregnancy BMI (< 25 kg/m2, ≥25 kg/m2), family history of diabetes (yes, no), baseline smoking status (never, ever), total physical activity (hours/week), race (white, non-white), education level (college graduate or higher, other), infertility diagnosis (male factor, female factor, unexplained), physician-diagnosed PCOS (yes, no), type of infertility treatment (IVF, IUI, natural), fetus number in a pregnancy (1, ≥2), and sex of fetus (es) (male, female or missing). † Test for linear trend were performed using the median SG-adjusted urinary BP-3 concentration in each quartile as a continuous variable in the model, adjusted for the above covariates. ‡ Adjusted p for interaction was obtained by adding interaction terms of season*the median SG-adjusted urinary BP-3 concentration in each quartile to the models described in †. § *p*-value for comparison against Q1 is < 0.05. **Table S4**. Quartiles of time-specific BP-3 urinary concentrations and blood glucose levels among pregnant women in the EARTH Study: stratified by maternal age. Legend: Abbreviations: LOD, limit of detection (0.4 ng/ml for some years and 0.2 ng/ml for other years). *Adjusted for pre-pregnancy BMI (< 25 kg/m2, ≥25 kg/m2), family history of diabetes (yes, no), baseline smoking status (never, ever), total physical activity (hours/week), race (white, non-white), education level (college graduate or higher, other), infertility diagnosis (male factor, female factor, unexplained), physician-diagnosed PCOS (yes, no), type of infertility treatment (IVF, IUI, natural), fetus number in a pregnancy (1, ≥2), sex of fetus (es) (male, female or missing), and season (spring, summer, fall, winter). † Test for linear trend were performed using the median SG-adjusted urinary BP-3 concentration in each quartile as a continuous variable in the model, adjusted for the above covariates. ‡ Adjusted p for interaction was obtained by adding interaction terms of maternal age*the median SG-adjusted urinary BP-3 concentration in each quartile to the models described in †. § *p*-value for comparison against Q1 is < 0.05. **Table S5**. Quartiles of time-specific BP-3 urinary concentrations and blood glucose levels among pregnant women in the EARTH Study: stratified by maternal BMI. Legend: Abbreviations: LOD, limit of detection (0.4 ng/ml for some years and 0.2 ng/ml for other years). * Adjusted for maternal age at pregnancy (years), family history of diabetes (yes, no), baseline smoking status (never, ever), total physical activity (hours/week), race (white, non-white), education level (college graduate or higher, other), infertility diagnosis (male factor, female factor, unexplained), physician-diagnosed PCOS (yes, no), type of infertility treatment (IVF, IUI, natural), fetus number in a pregnancy (1, ≥2), sex of fetus (es) (male, female or missing), and season (spring, summer, fall, winter). † Test for linear trend were performed using the median SG-adjusted urinary BP-3 concentration in each quartile as a continuous variable in the model, adjusted for the above covariates. ‡ Adjusted p for interaction was obtained by adding interaction terms of maternal BMI*the median SG-adjusted urinary BP-3 concentration in each quartile to the models described in †. § *p*-value for comparison against Q1 is < 0.05. **Table S6**. Quartiles of time-specific BP-3 urinary concentrations and blood glucose levels among pregnant women in the EARTH Study: stratified by sex of fetus (es). Legend: Abbreviations: LOD, limit of detection (0.4 ng/ml for some years and 0.2 ng/ml for other years). * Adjusted for maternal age at pregnancy (years), pre-pregnancy BMI (< 25 kg/m2, ≥25 kg/m2), family history of diabetes (yes, no), baseline smoking status (never, ever), total physical activity (hours/week), race (white, non-white), education level (college graduate or higher, other), infertility diagnosis (male factor, female factor, unexplained), physician-diagnosed PCOS (yes, no), type of infertility treatment (IVF, IUI, natural), fetus number in a pregnancy (1, ≥2), and season (spring, summer, fall, winter). † Test for linear trend were performed using the median SG-adjusted urinary BP-3 concentration in each quartile as a continuous variable in the model, adjusted for the above covariates. ‡ Adjusted p for interaction was obtained by adding interaction terms of sex of fetus (es)*the median SG-adjusted urinary BP-3 concentration in each quartile to the models described in †. § *p*-value for comparison against Q1 is < 0.05. **Figure S1**. SG-adjusted urinary BP-3 concentrations (in ng/mL) across seasons among 217 pregnant women in the EARTH Study. Legend: Overall p-value obtained from Type 3 analysis in linear regression, accounted for repeated measures of BP-3 concentrations within participants (number of participants = 217, number of samples = 833).

## Data Availability

The datasets in this study are not publicly available due to institutional restrictions prohibiting the sharing of data that contain information that could compromise research participant’s privacy upon which participant consent was contingent. However, the data are available from the corresponding author, Z.W., and the PI of the EARTH Study, R.H. upon reasonable request.

## Abbreviations

ART: Assisted reproductive technique
BMI: Body mass index
BP-3: Benzophenone-3
CDC: The Centers for Disease Control and Prevention
CI: Confidence interval
DM: Diabetes mellitus
EARTH: The Environment and Reproductive Health Study
EDC: Endocrine disrupting chemical
GDM: Gestational diabetes mellitus
GLT: Glucose load test
IGF-I: Insulin-like growth factor 1
IUI: Intrauterine insemination
IVF: In vitro fertilization
LOD: Limit of detection
MGH: Massachusetts General Hospital
NHANES: National Health and Nutrition Examination Survey
PCOS: Polycystic ovarian syndrome
POP: Persistent organic pollutant
Q1-Q4: Quartiles (1st to 4th)
SD: Standard deviation
SG: Specific gravity
T3: Triiodothyronine
UV: Ultraviolet
