# Supplementary material for: Perinatal urinary benzophenone-3 concentrations and glucose levels among women from a fertility clinic
Source: Environ Health. 2020 Apr 28;19:45. doi: 10.1186/s12940-020-00598-7 (PMC7189447; doi:10.1186/s12940-020-00598-7)
Supplement: Supplementary file 1 — Additional file 1: Table S1. Distribution of urinary BP-3 concentrations (ng/mL) among NHANES women during 2009–2016. Legend: Abbreviations: BP-3, benzophenone-3; LOD, limit of detection (0.4 ng/ml in NHANES 2009–2016). All estimates incorporated NHANES multistage sub-sample weights for combined survey cycles. BP-3 concentrations below LOD were assigned a value equal to the LOD divided by square root of 2 in NHANES.a Women in NHANES 2009–2016 who had positive lab pregnancy test results or self-reported pregnant at the time of urine sample collection, with available BP-3 concentrations. Due to disclosure risks, pregnancy status was only released for women 20–44 years of age since 2007.b Women in NHANES 2009–2016 who were 23–47 years old, regardless of pregnancy status. Table S2. Quartiles of time-specific BP-3 urinary concentrations and blood glucose levels among pregnant women in the EARTH Study: stratified by infertility diagnosis. Legend: Abbreviations: LOD, limit of detection (0.4 ng/ml for some years and 0.2 ng/ml for other years). *Adjusted for maternal age at pregnancy (years), pre-pregnancy BMI (< 25 kg/m2, ≥25 kg/m2), family history of diabetes (yes, no), baseline smoking status (never, ever), total physical activity (hours/week), race (white, non-white), education level (college graduate or higher, other), physician-diagnosed PCOS (yes, no), fetus number in a pregnancy (1, ≥2), sex of fetus (es) (male, female or missing) and season (spring, summer, fall, winter). † Test for linear trend were performed using the median SG-adjusted urinary BP-3 concentration in each quartile as a continuous variable in the model, adjusted for the above covariates. ‡ Adjusted p for interaction was obtained by adding interaction terms of infertility diagnosis*the median SG-adjusted urinary BP-3 concentration in each quartile to the models described in †. § p-value for comparison against Q1 is < 0.05. Table S3. Quartiles of time-specific BP-3 urinary concentrations and blood glu [file 12940_2020_598_MOESM1_ESM.doc]

**Additional file 1 - supplemental materials**

**Table S1**. Distribution of urinary BP-3 concentrations (ng/mL) among NHANES women during 2009-2016

|  | N | Detection % | Geometric mean  (95% CI) | Percentile | | | | | | |
| --- | --- | --- | --- | --- | --- | --- | --- | --- | --- | --- |
|  |  |  |  | Min | 25^th^ | 50^th^ | 75^th^ | 90^th^ | 95^th^ | Max |
| NHANES 2009-2016, pregnant women^a^ | 87 | 99.2% | 42.9  (23.9, 76.9) | <LOD | 9.9 | 28.8 | 120.7 | 1058.3 | 3396.0 | 7290.0 |
| NHANES 2009-2016, women aged 23-47 years^b^ | 1596 | 97.9% | 39.4  (32.9, 47.2) | <LOD | 8.0 | 32.1 | 161.7 | 876.9 | 2781.4 | 23641 |

Abbreviations: BP-3, benzophenone-3; LOD, limit of detection (0.4 ng/ml in NHANES 2009-2016).

All estimates incorporated NHANES multistage sub-sample weights for combined survey cycles.

BP-3 concentrations below LOD were assigned a value equal to the LOD divided by square root of 2 in NHANES.

*Women in NHANES 2009-2016 who had positive lab pregnancy test results or self-reported pregnant at the time of urine sample collection, with available BP-3 concentrations. Due to disclosure risks, pregnancy status was only released for women 20-44 years of age since 2007.

† Women in NHANES 2009-2016 who were 23-47 years old, regardless of pregnancy status.

**Table S2**. Quartiles of time-specific BP-3 urinary concentrations and blood glucose levels among pregnant women in the EARTH Study: stratified by infertility diagnosis

| Quartile (range) of SG-adjusted BP-3 concentration in ng/mL | Population means of blood glucose level in mg/dl (96% CI) across quartiles of SG-adjusted BP-3 | | | | | | | | |
| --- | --- | --- | --- | --- | --- | --- | --- | --- | --- |
|  | Male-factor | |  | | Female-factor | |  | Unexplained | |
|  | Unadjusted | Adjusted* |  | | Unadjusted | Adjusted* |  | Unadjusted | Adjusted* |
| **Urine samples collected during preconception (mean preconception BP-3 exposure for each participant)** | | | | | | | | | |
|  | N=50 | |  | | N=52 | |  | N=76 | |
| Q1  (<LOD to 52.6) | 116.3  (101.8, 132.7) | 119.7  (96.5, 148.6) |  | | 112.0  (99.2, 126.4) | 92.9  (81.0, 106.5) |  | 113.4  (102.6, 125.4) | 99.3  (84.7, 116.4) |
| Q2  (52.7 to 144.2) | 119.4  (104.0, 137.0) | 143.9  (116.3, 177.0) |  | | 121.5  (108.2, 136.3) | 94.7  (81.7, 109.9) |  | 106.3  (96.3, 117.2) | 96.3  (79.8, 116.2) |
| Q3  (144.4 to 463.6) | 121.8  (105.6, 140.6) | 114.6  (92.3, 142.3) |  | | 103.3  (94.0, 113.5) | 80.9  (71.4, 91.7) § |  | 117.0  (104.5, 131.0) | 99.2  (81.6, 120.5) |
| Q4  (467.7 to 7794.2) | 109.1  (94.0, 126.7) | 122.1  (96.5, 154.5) |  | | 101.2  (89.7, 114.2) | 82.8  (72.4, 94.7) |  | 114.0  (103.8, 125.2) | 101.0  (83.7, 121.8) |
| *P*-trend† | 0.58 | 0.81 |  | | 0.09 | 0.06 |  | 0.66 | 0.64 |
| Adjusted p for interaction‡ *(Male factor vs. Female factor)* | | | | 0.30 | | | | | |
| Adjusted p for interaction‡ (*Female factor vs. Unexplained*) | | | | 0.04 | | | | | |
| Adjusted p for interaction‡ *(Male factor vs. Unexplained)* | | | | 0.35 | | | | | |
| Adjusted p for interaction‡ *(Joint test)* | | | | 0.12 | | | | | |
| **Urine samples collected during first trimester (one sample per participant)** | | | | | | | | | |
|  | N=53 | |  | | N=60 | |  | N=81 | |
| Q1  (<LOD to 33.0) | 123.2  (108.7, 139.7) | 127.6  (105.7, 154.0) |  | | 119.6  (106.3, 134.5) | 108.5  (93.2, 126.2) |  | 109.4  (99.3, 120.4) | 97.8  (86.2, 111.0) |
| Q2  (36.5 to 116.3) | 121.5  (107.9, 136.7) | 129.9  (102.5, 164.5) |  | | 106.6  (95.9, 118.4) | 96.6  (85.4, 109.3) |  | 109.8  (98.6, 122.3) | 104.4  (90.9, 119.9) |
| Q3  (117.5 to 471.2) | 124.7  (108.5, 143.2) | 123.6  (97.9, 156.1) |  | | 113.7  (101.6, 127.3) | 102.2  (87.5, 119.4) |  | 123.9  (113.3, 135.6) | 116.5  (102.9, 131.8) § |
| Q4  (480.0 to 7395.0) | 94.1  (76.7, 115.5) | 106.0  (83.9, 135.5) |  | | 99.5  (90.9, 109.0) | 87.2  (77.5, 98.0) § |  | 112.3  (101.5, 122.0) | 101.2  (88.8, 115.5) |
| *P*-trend† | 0.12 | 0.18 |  | | 0.03 | 0.01 |  | 0.53 | 0.44 |
| Adjusted p for interaction‡ *(Male factor vs. Female factor)* | | | | 0.96 | | | | | |
| Adjusted p for interaction‡ (*Female factor vs. Unexplained*) | | | | 0.02 | | | | | |
| Adjusted p for interaction‡ *(Male factor vs. Unexplained)* | | | | 0.04 | | | | | |
| Adjusted p for interaction‡ *(Joint test)* | | | | 0.03 | | | | | |
| **Urine samples collected during second trimester (one sample per participant)** | | | | | | | | | |
|  | N=48 | |  | | N=49 | |  | N=73 | |
| Q1  (<LOD to 36.9) | 115.4  (102.3, 130.2) | 128.0  (107.4, 152.5) |  | | 121.4  (107.3, 137.3) | 109.3  (92.7, 128.8) |  | 113.2  (102.0, 125.6) | 93.5  (80.2, 109.0) |
| Q2  (37.6 to 157.1) | 103.6  (87.8, 122.3) | 111.8  (87.5, 142.7) |  | | 107.8  (98.0, 118.6) | 101.1  (86.8, 117.8) |  | 108.5  (98.6, 119.4) | 93.5  (80.1, 109.1) |
| Q3  (160.0 to 761.1) | 114.2  (98.9, 131.8) | 129.8  (105.8, 159.2) |  | | 99.3  (89.2, 110.5) | 89.6  (77.0, 104.2) § |  | 109.3  (99.4, 120.3) | 90.7  (77.5, 106.2) |
| Q4  (801.8 to 16296.7) | 130.2  (111.3, 152.4) | 143.0  (114.1, 179.3) |  | | 103.5  (93.4, 114.7) | 88.1  (76.6, 101.3) § |  | 111.3  (101.2, 122.5) | 87.1  (74.2, 102.3) |
| *P*-trend† | 0.26 | 0.32 |  | | 0.06 | 0.005 |  | 0.90 | 0.25 |
| Adjusted p for interaction‡ *(Male factor vs. Female factor)* | | | | 0.02 | | | | | |
| Adjusted p for interaction‡ (*Female factor vs. Unexplained*) | | | | 0.13 | | | | | |
| Adjusted p for interaction‡ *(Male factor vs. Unexplained)* | | | | 0.36 | | | | | |
| Adjusted p for interaction‡ *(Joint test)* | | | | 0.08 | | | | | |

Abbreviations: LOD, limit of detection (0.4 ng/ml for some years and 0.2 ng/ml for other years)

*Adjusted for maternal age at pregnancy (years), pre-pregnancy BMI (<25 kg/m^2^, ≥25 kg/m^2^), family history of diabetes (yes, no), baseline smoking status (never, ever), total physical activity (hours/week), race (white, non-white), education level (college graduate or higher, other), physician-diagnosed PCOS (yes, no), fetus number in a pregnancy (1, ≥2), sex of fetus(es) (male, female or missing) and season (spring, summer, fall, winter).

† Test for linear trend were performed using the median SG-adjusted urinary BP-3 concentration in each quartile as a continuous variable in the model, adjusted for the above covariates.

‡ Adjusted p for interaction was obtained by adding interaction terms of infertility diagnosis*the median SG-adjusted urinary BP-3 concentration in each quartile to the models described in †.

§ p-value for comparison against Q1 is < 0.05.

**Table S3. Quartiles of time-specific BP-3 urinary concentrations and blood glucose levels among pregnant women in the EARTH Study: stratified by season of urine collection**

| Quartile (range) of SG-adjusted BP-3 concentration in ng/mL | Population means of blood glucose level in mg/dl (95% CI) across quartiles of SG-adjusted BP-3 | | | | |
| --- | --- | --- | --- | --- | --- |
|  | Summer | |  | Other seasons | |
|  | Unadjusted | Adjusted* |  | Unadjusted | Adjusted* |
| **Urine samples collected during preconception (mean preconception BP-3 exposure for each participant)** | | | | | |
|  | N=39 | |  | N=139 | |
| Q1 (<LOD to 52.6) | 132.7 (114.0, 154.4) | 137.1 (115.6, 162.7) |  | 109.6 (101.6, 118.2) | 106.6 (97.6, 116.5) |
| Q2 (52.7 to 144.2) | 113.4 (92.5, 139.0) | 130.2 (99.5, 170.2) |  | 113.9 (106.2, 122.3) | 110.6 (100.3, 121.9) |
| Q3 (144.4 to 463.6) | 108.8 (95.9, 123.5) | 101.0 (79.2, 128.9) § |  | 114.0 (105.4, 123.4) | 104.4 (93.9, 116.2) |
| Q4 (467.7 to 7794.2) | 101.4 (88.9, 115.6) | 100.7 (86.9, 116.6) § |  | 112.7 (104.1, 121.9) | 110.0 (98.7, 122.5) |
| *P*-trend† | 0.01 | <0.0001 |  | 0.67 | 0.80 |
| Adjusted p for interaction‡ | 0.005 | | | | |
| **Urine samples collected during first trimester (one sample per participant)** | | | | | |
|  | N=43 | |  | N=151 | |
| Q1 (<LOD to 33.0) | 107.6 (93.7, 123.4) | 89.2 (74.4, 107.1) |  | 117.9 (109.5, 127.1) | 117.4 (107.5, 128.2) |
| Q2 (36.5 to 116.3) | 114.6 (101.6, 129.4) | 104.7 (88.7, 123.5) |  | 112.5 (104.4, 121.4) | 109.8 (99.1, 121.7) |
| Q3 (117.5 to 471.2) | 123.4 (111.6, 136.5) | 120.6 (104.0, 139.9) § |  | 120.6 (111.4, 130.6) | 116.3 (104.9, 128.9) |
| Q4 (480.0 to 7395.0) | 99.7 (90.5, 109.9) | 87.9 (73.3, 105.4) |  | 105.9 (97.5, 114.9) | 105.8 (96.0, 116.6) |
| *P*-trend† | 0.28 | 0.83 |  | 0.14 | 0.10 |
| Adjusted p for interaction‡ | 0.61 | | | | |
| **Urine samples collected during second trimester (one sample per participant)** | | | | | |
|  | N=53 | |  | N=117 | |
| Q1 (<LOD to 36.9) | 123.2 (106.1, 143.1) | 125.3 (102.6, 153.1) |  | 113.5 (105.4, 122.2) | 103.6 (93.8, 114.3) |
| Q2 (37.6 to 157.1) | 117.7 (101.4, 136.7) | 114.2 (93.8, 139.1) |  | 104.2 (96.9, 112.1) | 101.0 (90.6, 112.6) |
| Q3 (160.0 to 761.1) | 104.6 (92.6, 118.2) | 99.1 (83.4, 117.8) § |  | 109.4 (101.1, 118.4) | 100.9 (89.8, 113.2) |
| Q4 (801.8 to 16296.7) | 111.5 (99.8, 124.7) | 107.9 (92.9, 125.2) |  | 114.1 (104.7, 124.2) | 102.9 (91.6, 115.6) |
| *P*-trend† | 0.25 | 0.12 |  | 0.79 | 0.87 |
| Adjusted p for interaction‡ | 0.18 | | | | |

Abbreviations: LOD, limit of detection (0.4 ng/ml for some years and 0.2 ng/ml for other years)

*Adjusted for maternal age at pregnancy (years), pre-pregnancy BMI (<25 kg/m^2^, ≥25 kg/m^2^), family history of diabetes (yes, no), baseline smoking status (never, ever), total physical activity (hours/week), race (white, non-white), education level (college graduate or higher, other), infertility diagnosis (male factor, female factor, unexplained), physician-diagnosed PCOS (yes, no), type of infertility treatment (IVF, IUI, natural), fetus number in a pregnancy (1, ≥2), and sex of fetus(es) (male, female or missing).

† Test for linear trend were performed using the median SG-adjusted urinary BP-3 concentration in each quartile as a continuous variable in the model, adjusted for the above covariates.

‡ Adjusted p for interaction was obtained by adding interaction terms of season*the median SG-adjusted urinary BP-3 concentration in each quartile to the models described in †.

§ p-value for comparison against Q1 is < 0.05.

**Table S4**. Quartiles of time-specific BP-3 urinary concentrations and blood glucose levels among pregnant women in the EARTH Study: stratified by maternal age

| Quartile (range) of SG-adjusted BP-3 concentration in ng/mL | Population means of blood glucose level in mg/dl (95% CI) across quartiles of SG-adjusted BP-3 | | | | |
| --- | --- | --- | --- | --- | --- |
|  | Maternal age < 37 | |  | Maternal age ≥ 37 | |
|  | Unadjusted | Adjusted* |  | Unadjusted | Adjusted* |
| **Urine samples collected during preconception (mean preconception BP-3 exposure for each participant)** | | | | | |
|  | N=126 | |  | N=52 | |
| Q1 (<LOD to 52.6) | 108.3 (99.4, 118.1) | 108.0 (98.4, 118.6) |  | 125.6 (113.4, 139.1) | 136.7 (118.8, 157.2) |
| Q2 (52.7 to 144.2) | 109.4 (101.0, 118.5) | 110.6 (99.7, 122.7) |  | 128.8 (114.3, 145.1) | 141.4 (119.3, 167.4) |
| Q3 (144.4 to 463.6) | 111.2 (102.3, 120.9) | 106.1 (95.2, 118.1) |  | 115.5 (103.9, 128.4) | 123.0 (103.5, 146.2) |
| Q4 (467.7 to 7794.2) | 110.2 (101.5, 119.7) | 108.8 (97.6, 121.2) |  | 107.4 (95.8, 120.4) | 122.4 (105.5, 142.1) |
| *P*-trend† | 0.75 | 0.96 |  | 0.03 | 0.04 |
| Adjusted p for interaction‡ | 0.14 | | | | |
| **Urine samples collected during first trimester (one sample per participant)** | | | | | |
|  | N=135 | |  | N=59 | |
| Q1 (<LOD to 33.0) | 111.6 (102.8, 121.2) | 110.3 (100.8, 120.6) |  | 126.4 (114.0, 140.1) | 125.1 (106.9, 146.3) |
| Q2 (36.5 to 116.3) | 111.1 (102.5, 120.4) | 109.3 (98.8, 121.1) |  | 116.8 (105.3, 129.4) | 120.2 (104.5, 138.3) |
| Q3 (117.5 to 471.2) | 122.7 (113.6, 132.6) | 121.4 (110.2, 133.6) § |  | 117.5 (104.8, 131.7) | 109.4 (93.8, 127.7) |
| Q4 (480.0 to 7395.0) | 99.8 (92.2, 108.0) | 98.9 (89.7, 109.0) |  | 115.1 (103.1, 128.5) | 118.1 (103.1, 135.2) |
| *P*-trend† | 0.16 | 0.17 |  | 0.24 | 0.20 |
| Adjusted p for interaction‡ | 0.85 | | | | |
| **Urine samples collected during second trimester (one sample per participant)** | | | | | |
|  | N=113 | |  | N=57 | |
| Q1 (<LOD to 36.9) | 110.7 (101.3, 121.0) | 105.3 (95.2, 116.5) |  | 125.5 (114.0, 138.1) | 123.6 (105.3, 145.2) |
| Q2 (37.6 to 157.1) | 108.4 (98.8, 119.0) | 107.8 (95.7, 121.4) |  | 105.5 (96.7, 115.2) | 102.2 (86.6, 120.6) § |
| Q3 (160.0 to 761.1) | 105.3 (96.3, 115.1) | 100.5 (89.6, 112.7) |  | 111.9 (102.0, 122.8) | 111.7 (98.3, 126.9) |
| Q4 (801.8 to 16296.7) | 111.3 (102.8, 120.6) | 103.4 (93.4, 114.3) |  | 120.2 (105.4, 137.1) | 109.0 (92.0, 129.1) |
| *P*-trend† | 0.96 | 0.56 |  | 0.57 | 0.08 |
| Adjusted p for interaction‡ | 0.46 | | | | |

Abbreviations: LOD, limit of detection (0.4 ng/ml for some years and 0.2 ng/ml for other years)

*Adjusted for pre-pregnancy BMI (<25 kg/m^2^, ≥25 kg/m^2^), family history of diabetes (yes, no), baseline smoking status (never, ever), total physical activity (hours/week), race (white, non-white), education level (college graduate or higher, other), infertility diagnosis (male factor, female factor, unexplained), physician-diagnosed PCOS (yes, no), type of infertility treatment (IVF, IUI, natural), fetus number in a pregnancy (1, ≥2), sex of fetus(es) (male, female or missing), and season (spring, summer, fall, winter).

† Test for linear trend were performed using the median SG-adjusted urinary BP-3 concentration in each quartile as a continuous variable in the model, adjusted for the above covariates.

‡ Adjusted p for interaction was obtained by adding interaction terms of maternal age*the median SG-adjusted urinary BP-3 concentration in each quartile to the models described in †.

§ p-value for comparison against Q1 is < 0.05.

**Table S5**. Quartiles of time-specific BP-3 urinary concentrations and blood glucose levels among pregnant women in the EARTH Study: stratified by maternal BMI

| Quartile (range) of SG-adjusted BP-3 concentration in ng/mL | Population means of blood glucose level in mg/dl (95% CI) across quartiles of SG-adjusted BP-3 | | | | |
| --- | --- | --- | --- | --- | --- |
|  | Maternal BMI < 25 | |  | Maternal BMI ≥ 25 | |
|  | Unadjusted | Adjusted* |  | Unadjusted | Adjusted* |
| **Urine samples collected during preconception (mean preconception BP-3 exposure for each participant)** | | | | | |
|  | N=121 | |  | N=57 | |
| Q1 (<LOD to 52.6) | 107.5 (98.9, 116.8) | 104.4 (92.8, 117.4) |  | 125.1 (111.7, 140.1) | 110.5 (95.3, 128.1) |
| Q2 (52.7 to 144.2) | 113.8 (105.8, 122.5) | 109.7 (97.3, 123.7) |  | 114.0 (98.3, 132.2) | 115.1 (96.4, 137.3) |
| Q3 (144.4 to 463.6) | 106.8 (98.4, 116.0) | 99.1 (86.7, 113.2) |  | 122.5 (109.3, 137.2) | 118.2 (101.1, 138.2) |
| Q4 (467.7 to 7794.2) | 106.5 (98.5, 115.1) | 101.0 (88.9, 114.7) |  | 117.0 (102.6, 133.1) | 111.5 (95.3, 128.1) |
| *P*-trend† | 0.57 | 0.32 |  | 0.56 | 0.75 |
| Adjusted p for interaction‡ | 0.35 | | | | |
| **Urine samples collected during first trimester (one sample per participant)** | | | | | |
|  | N=127 | |  | N=67 | |
| Q1 (<LOD to 33.0) | 110.6 (102.3, 119.6) | 108.3 (95.8, 122.5) |  | 127.6 (114.4, 142.3) | 116.0 (103.0, 130.5) |
| Q2 (36.5 to 116.3) | 112.6 (104.3, 121.7) | 109.8 (96.7, 124.6) |  | 113.4 (101.7, 126.5) | 111.8 (98.4, 127.0) |
| Q3 (117.5 to 471.2) | 116.9 (108.3, 126.3) | 113.6 (101.0, 127.9) |  | 130.1 (116.6, 145.1) | 120.4 (104.5, 138.7) |
| Q4 (480.0 to 7395.0) | 97.8 (91.1, 105.6) | 94.8 (83.8, 107.2) § |  | 117.7 (105.1, 131.7) | 112.9 (98.7, 129.1) |
| *P*-trend† | 0.05 | 0.03 |  | 0.60 | 0.94 |
| Adjusted p for interaction‡ | 0.20 | | | | |
| **Urine samples collected during second trimester (one sample per participant)** | | | | | |
|  | N=112 | |  | N=58 | |
| Q1 (<LOD to 36.9) | 109.0 (100.0, 118.7) | 101.4 (85.0, 120.9) |  | 127.8 (115.6, 141.2) | 116.9 (104.2, 131.1) |
| Q2 (37.6 to 157.1) | 106.0 (98.4, 114.1) | 98.8 (84.2, 115.9) |  | 112.8 (97.9, 129.9) | 100.7 (85.7, 118.3) |
| Q3 (160.0 to 761.1) | 109.7 (101.0, 119.1) | 100.7 (84.6, 119.8) |  | 104.1 (93.9, 115.5) | 97.5 (86.0, 110.6) § |
| Q4 (801.8 to 16296.7) | 104.0 (95.0, 113.9) | 91.7 (76.4, 109.9) |  | 124.8 (113.9, 136.9) | 118.7 (104.9, 134.4) |
| *P*-trend† | 0.63 | 0.17 |  | 0.67 | 0.96 |
| Adjusted p for interaction‡ | 0.37 | | | | |

Abbreviations: LOD, limit of detection (0.4 ng/ml for some years and 0.2 ng/ml for other years)

* Adjusted for maternal age at pregnancy (years), family history of diabetes (yes, no), baseline smoking status (never, ever), total physical activity (hours/week), race (white, non-white), education level (college graduate or higher, other), infertility diagnosis (male factor, female factor, unexplained), physician-diagnosed PCOS (yes, no), type of infertility treatment (IVF, IUI, natural), fetus number in a pregnancy (1, ≥2), sex of fetus(es) (male, female or missing), and season (spring, summer, fall, winter).

† Test for linear trend were performed using the median SG-adjusted urinary BP-3 concentration in each quartile as a continuous variable in the model, adjusted for the above covariates.

‡ Adjusted p for interaction was obtained by adding interaction terms of maternal BMI*the median SG-adjusted urinary BP-3 concentration in each quartile to the models described in †.

§ p-value for comparison against Q1 is < 0.05.

**Table S6**. Quartiles of time-specific BP-3 urinary concentrations and blood glucose levels among pregnant women in the EARTH Study: stratified by sex of fetus(es)

| Quartile (range) of SG-adjusted BP-3 concentration in ng/mL | Population means of blood glucose level in mg/dl (95% CI) across quartiles of SG-adjusted BP-3 | | | | |
| --- | --- | --- | --- | --- | --- |
|  | Male fetus(es) | |  | Female fetus(es) | |
|  | Unadjusted | Adjusted* |  | Unadjusted | Adjusted* |
| **Urine samples collected during preconception (mean preconception BP-3 exposure for each participant)** | | | | | |
|  | N=84 | |  | N=91 | |
| Q1 (<LOD to 52.6) | 112.3 (101.5, 124.2) | 107.0 (95.4, 120.1) |  | 115.5 (105.2, 126.8) | 109.9 (97.6, 123.8) |
| Q2 (52.7 to 144.2) | 114.5 (103.8, 126.4) | 99.3 (86.2, 114.3) |  | 114.9 (104.2, 126.7) | 113.8 (99.0, 130.8) |
| Q3 (144.4 to 463.6) | 110.6 (100.8, 121.3) | 99.7 (87.3, 114.0) |  | 115.0 (104.0, 127.1) | 104.2 (90.7, 119.6) |
| Q4 (467.7 to 7794.2) | 115.6 (103.0, 129.8) | 103.1 (89.4, 118.8) |  | 106.6 (97.8, 116.2) | 100.2 (87.9, 114.3) |
| *P*-trend† | 0.84 | 0.59 |  | 0.19 | 0.08 |
| Adjusted p for interaction‡ | 0.56 | | | | |
| **Urine samples collected during first trimester (one sample per participant)** | | | | | |
|  | N=91 | |  | N=100 | |
| Q1 (<LOD to 33.0) | 106.4 (96.1, 117.9) | 107.8 (94.2, 123.4) |  | 124.7 (114.9, 135.4) | 122.3 (110.4, 135.4) |
| Q2 (36.5 to 116.3) | 117.8 (106.8, 129.9) | 111.7 (96.7, 129.1) |  | 108.8 (100.0, 118.2) § | 109.5 (98.4, 121.7) § |
| Q3 (117.5 to 471.2) | 124.1 (112.0, 137.4) § | 110.8 (95.4, 128.7) |  | 121.2 (111.5, 131.7) | 121.7 (109.1, 135.8) |
| Q4 (480.0 to 7395.0) | 104.9 (95.6, 115.0) | 103.2 (90.6, 117.5) |  | 103.5 (94.3, 113.6) § | 100.6 (89.8, 112.7) § |
| *P*-trend† | 0.80 | 0.47 |  | 0.02 | 0.009 |
| Adjusted p for interaction‡ | 0.25 | | | | |
| **Urine samples collected during second trimester (one sample per participant)** | | | | | |
|  | N=81 | |  | N=87 | |
| Q1 (<LOD to 36.9) | 116.1 (105.5, 127.9) | 111.3 (99.2, 125.0) |  | 115.4 (105.0, 127.0) | 111.9 (98.4, 127.2) |
| Q2 (37.6 to 157.1) | 107.4 (97.6, 118.3) | 99.3 (86.5, 114.0) |  | 107.0 (97.5, 117.4) | 106.6 (93.6, 121.4) |
| Q3 (160.0 to 761.1) | 105.8 (96.3, 116.2) | 101.9 (89.5, 116.1) |  | 111.3 (101.0, 122.7) | 106.6 (94.6, 120.1) |
| Q4 (801.8 to 16296.7) | 115.0 (104.5, 126.7) | 106.4 (93.3, 121.3) |  | 112.0 (101.6, 123.5) | 103.4 (91.1, 117.2) |
| *P*-trend† | 0.88 | 0.49 |  | 0.83 | 0.28 |
| Adjusted p for interaction‡ | 0.98 | | | | |

Abbreviations: LOD, limit of detection (0.4 ng/ml for some years and 0.2 ng/ml for other years)

*Adjusted for maternal age at pregnancy (years), pre-pregnancy BMI (<25 kg/m^2^, ≥25 kg/m^2^), family history of diabetes (yes, no), baseline smoking status (never, ever), total physical activity (hours/week), race (white, non-white), education level (college graduate or higher, other), infertility diagnosis (male factor, female factor, unexplained), physician-diagnosed PCOS (yes, no), type of infertility treatment (IVF, IUI, natural), fetus number in a pregnancy (1, ≥2), and season (spring, summer, fall, winter).

† Test for linear trend were performed using the median SG-adjusted urinary BP-3 concentration in each quartile as a continuous variable in the model, adjusted for the above covariates.

‡ Adjusted p for interaction was obtained by adding interaction terms of sex of fetus(es)*the median SG-adjusted urinary BP-3 concentration in each quartile to the models described in †.

§ p-value for comparison against Q1 is < 0.05.


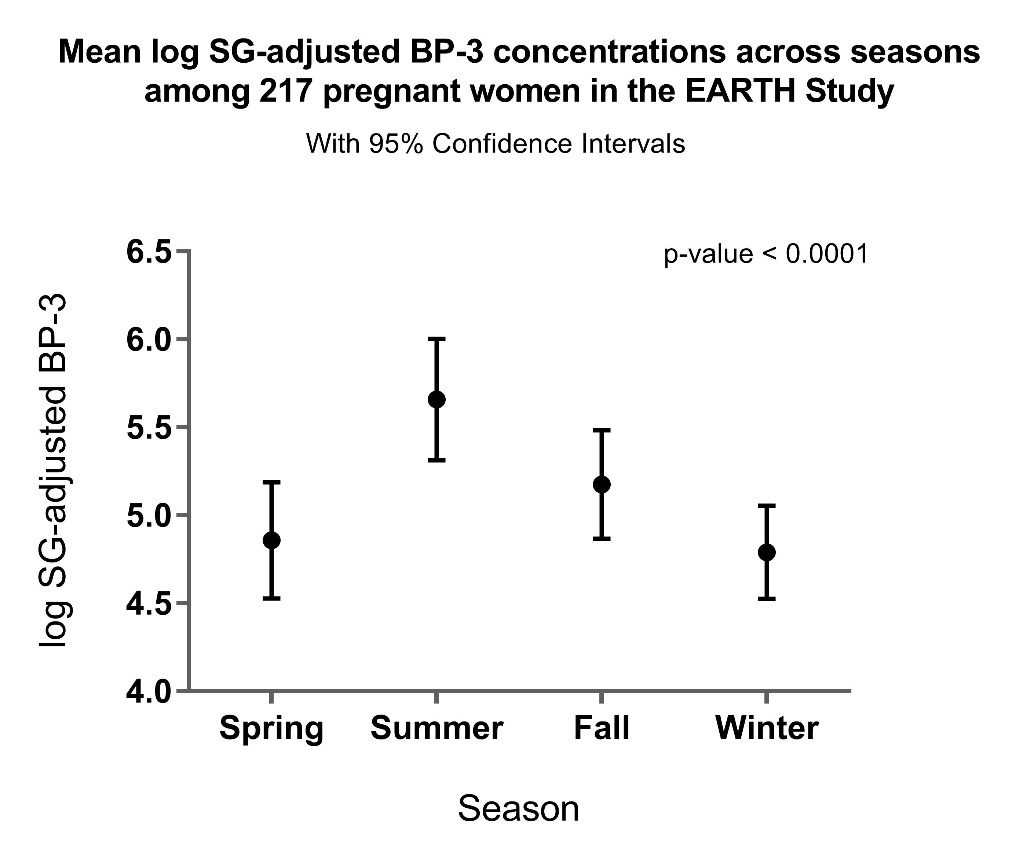


**Figure S1.** SG-adjusted urinary BP-3 concentrations (in ng/mL) across seasons among 217 pregnant women in the EARTH Study.

Overall p-value obtained from Type 3 analysis in linear regression, accounted for repeated measures of BP-3 concentrations within participants (number of participants = 217, number of samples = 833).

Seasons: winter (December, January, February), spring (March, April, May), summer (June, July, August), fall (September, October, November).
